# Musical aptitude moderates ease and vividness but not frequency of the speech-to-song illusion

**DOI:** 10.1038/s41598-025-18612-8

**Published:** 2025-09-26

**Authors:** Tamara V. Rathcke, Eline A. Smit

**Affiliations:** Department of Linguistics, University of Konstanz, 78464 Konstanz, Germany

**Keywords:** Human behaviour, Prognostic markers

## Abstract

Repetitions of a spoken phrase can induce a perceptual illusion in which speech transforms into song, known as the speech-to-song illusion. Speech acoustics that share certain pitch and timing properties with songs seem to be involved in facilitating the illusion, with a recent proposal suggesting that the illusion hinges on the individual ability to detect musical features latently present in speech. The current study tests this proposal by manipulating pitch and timing features of spoken phrases and examining how musical aptitude of listeners (specifically, their sensitivity to disruptions of musical melody and beat timing) moderates their experience of the illusion. The results show that the illusion is perceived by everyone regardless of their musical aptitude, with phrases that contain stable pitch and long periods of high sonority transforming more frequently. However, musical aptitude does moderate the speed and the strength of the illusion. Listeners with lower beat perception ability experience the illusion faster, which suggests involvement of temporal distortion processes during repetitions. Listeners with higher melody perception ability experience the illusion more strongly, which indicates involvement of musical pitch extraction rather than pitch distortion. These findings contribute new evidence on the complexity of an illusory experience in the auditory domain.

## Introduction

The phenomenon known as the speech-to-song (STS) illusion^1,2^ describes a perceptual experience of speech transforming into song upon repetition. It documents a striking discrepancy between the physical signal that encodes speech and the subjective perception that decodes song, demonstrating a link between two cognitive systems of the human mind—language and music. Being sound-based, performed music and spoken language share the medium of acoustic transmission. However, the physical structure of the two expressive systems—at least in Western countries—typically differs between language and music, with little overlap in the acoustic representations of speech and song^3^. Deep neural network classifiers can be reliably trained to automatically distinguish between speech and song with a high level of accuracy^4^. Young children acquire the core knowledge about representational differences between speech and song by the age of four and demonstrate adult-like performance in the categorisation of the two types of acoustic signals by the age of eight^5^. Adult listeners are able to tell music from speech within the first 25 ms of hearing the sound^6^. But under certain circumstances, the mind seems to abandon the perceptual distinction between speech and song and experiences STS.

The illusion was first discovered and coined by Diana Deutsch^1^ who pointed out that *“this effect is not just one of interpretation, since listeners upon hearing several repetitions of the phrase sing it back with the pitches distorted so as to give rise to a well-formed melody*^2^*”.* The transformation typically occurs after the third repetition of a spoken phrase^7^ and tends to persist, unable to be “unheard”, leading to a continued perception of a song and a melody (even after the repetitions have stopped)^8^. STS is accompanied by an alteration in the involved neural circuits, increasingly recruiting the regions associated with pitch extraction, song production, and auditory-motor integration^9^. Repetition is key to the experience of the illusion. Not only speech, but also environmental sounds and tonal sequences tend to be perceived as musical after repetitions^10,11^. It has been argued that repetition activates an internal mechanism that sets a perceptual bias for music^10,11^, possibly because repetition is an integral part of (Western style) music^12^.

Recent evidence suggests that a bias toward the perception of musical structure may also feed from other sources. For example, syntactic structure and meaning of spoken phrases can have a profound effect on whether or not a spoken phrase gives rise to STS^13^. If a phrase expresses an implausible meaning (e.g., *Trains can fly*.) or contains an implausible syntactic parse (e.g., *Every time Sue laughs a glass, shatters*.), it tends to ignite the transformation into song more often and leads to a more vivid STS-experience than a spoken phrase with plausible meaning (e.g., *Ducks can fly*.) and syntactic parse (e.g., *Every time Tom laughs, a glass bursts.*)^13^. Given that violations of semantic plausibility^14^ and syntactic parsing constraints^15,16^ are commonly found in (song) lyrics, it is not unlikely that a bias toward the perception of musical structure can be shaped by a variety of casual daily experiences with listening to music and songs^17^, equipping listeners with implicit knowledge about different aspects of their music culture—we call them music-related priors—and hereby creating the basis for a perceptual bias toward song^18^.

A major bias toward the perception of musical structure is, however, of acoustic origin. Naturally varying acoustics of speech—primarily its prosodic aspects such as pitch and rhythm—have been shown to influence the likelihood, ease, and/or strength of the illusion^7,19–21^. Specifically, local pitch stability (as opposed to more dynamic pitch trajectories) enhances the experience of STS^7,8,20^. In contrast, scalar musical pitch intervals seem to have very little effect on STS^7,20^, supporting the suggestion that the illusion leads to a perceptual creation of a musical melody without strong scalar cues being present in the acoustics of the spoken phrase^1,2^. Moreover, the illusion prevails in spoken phrases whose phonological structure and resulting levels of sonority make pitch information easier to extract^19^. Variability in local timing of inter-vocalic (as opposed to inter-syllabic) intervals also promotes the illusion^7^ while more global rhythmic features such as accentual regularity within the phrase^7,20^ or across phrase repetitions^7,22^ do not affect the experience of STS. These findings suggest that, at least to some extent, the experience of the illusion is driven by the acoustics of spoken phrases, some of which naturally contain properties that can be more readily perceived as musical upon repetition^7,20,23^. Indeed, it has been suggested that the experience of the illusion crucially hinges on the individual perceptual sensitivity to musical characteristics latent in speech acoustics^23^. Individual musical aptitude—specifically the ability to attend exclusively to pitch and to track beat and tonality misalignments—has been shown to correlate with the strength of individual experience of STS^23^. Such individual tendency to prioritise pitch information in speech can also arise from an individually variable reliance on prosody for syntactic computations during spoken language processing^24–26^ and also tends to facilitate the experience of the illusion^13^.

Individual variability in experiencing STS is relatively large^19,23^, and its interaction with different features of speech acoustics during the exposure to spoken phrase repetitions is, so far, poorly understood. Moreover, research to date has rarely examined the three distinct aspects of the illusion: (1) whether or not listeners can experience STS, i.e., the likelihood of the illusion; (2) the ease with which the transformation occurs for them, i.e., the speed of the illusion; and (3) how vividly song-like the phrase sounds to them after repetitions, i.e., the strength of the illusion^13^. Previous findings demonstrate that these aspects of the illusion are partly dissociable^13^, and may therefore differ with regards to the involvement of individual musical aptitude traits and the role of the acoustic-prosodic aspects of spoken phrases. The understanding of how such individual listener traits may interact with phrasal pitch and timing in shaping different aspects of STS is central to theoretical models of the illusion that propose detection of musical features in spoken input to be the key mechanism giving rise to STS^23^.

The present study was conducted to examine how the three aspects of STS may be influenced by individual sensitivity to disruptions of pitch (here, exemplified by mistuning of musical melodies)^27^ and timing (here, examplified by misalignment of musical beats)^28^ and their interaction with pitch and timing fluctuations of spoken phrases. For this, we recruited 86 native English listeners who completed two psychometric tests of musical aptitude: (1) the Computerised Adaptive Beat Alignment Test (CA-BAT)^28^ which measured participants’ beat perception ability as individual sensitivity to temporal misalignments of the beat and (2) the Mistuning Perception Test (MPT)^27^ which measured participants’ melody perception ability as individual sensitivity to vocal mistuning. In addition, participants performed a speech-to-song illusion test, listening to repetitions of English phrases and responding when they experienced a transformation from speech to song and how strongly song-like a phrase sounded to them, or reporting that they did not perceive the transformation^7,13,19^. The phrases that listeners had to judge were taken from a previous study^19^ which documented that these specific phrases were low-transforming on all three aspects of the illusion. We manipulated two acoustic-prosodic characteristics of these phrases to increase their transformability, by either flattening local pitch trajectories (pitch-manipulated phrases) or by lengthening the sonorous part and shortening the obstruent part of each syllable while keeping the total phrase duration constant (duration-manipulated phrases). Following on from the proposals suggesting that experience of STS is based on the perception of musical features inherent to spoken phrases and is modertated by individual musical aptitude^23^, we hypothesised that listeners with high melody perception ability would experience STS more frequently, faster, and more vividly especially in pitch-manipulated phrases while listeners with high beat perception ability would show a heightened experience of STS in duration-manipulated stimuli.

## Results

### Relationship between individual melody and beat perception abilities

**Fig. 1:**
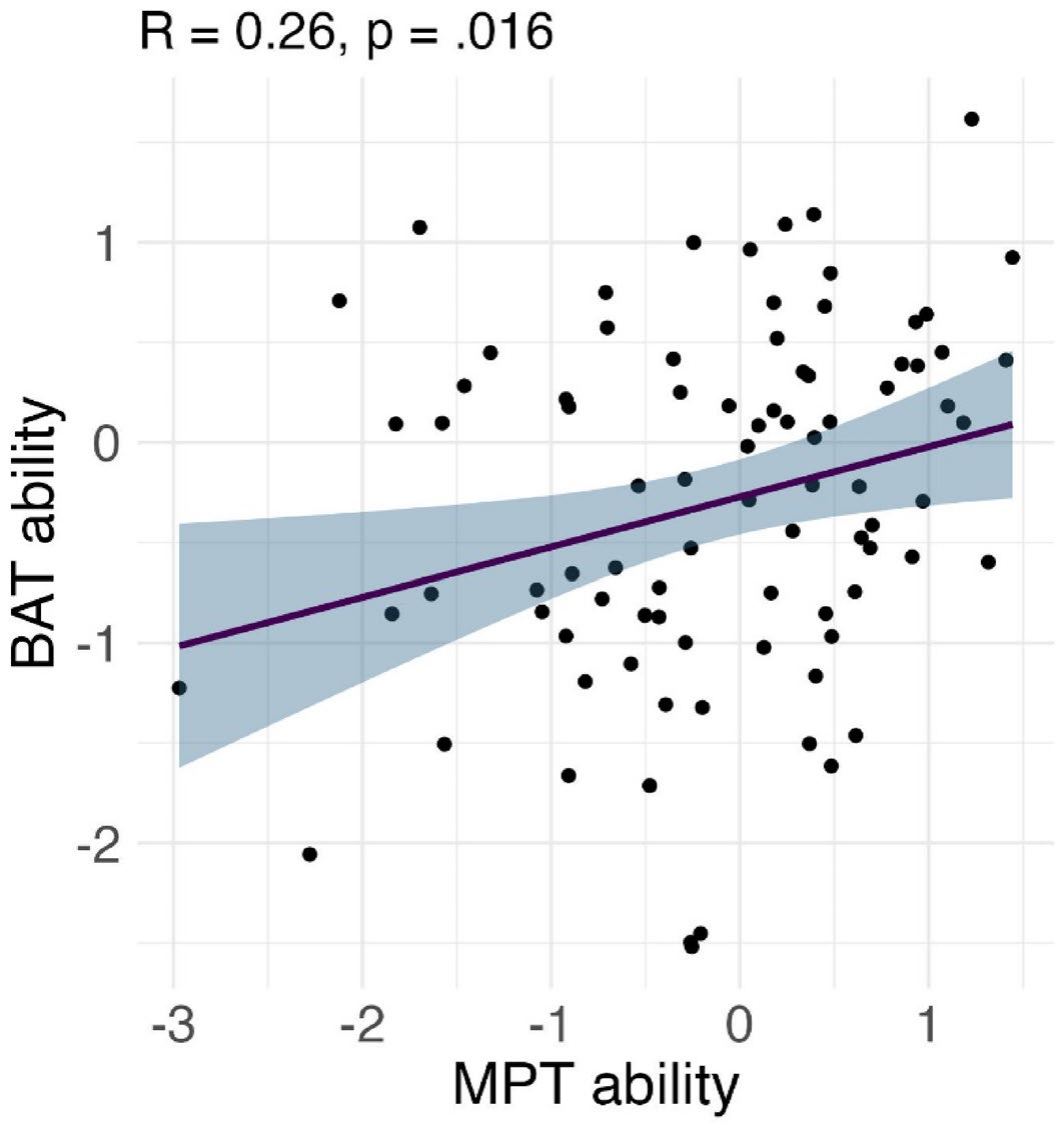
Correlation between indices of individual melody and beat perception assessed by the Mistuning Perception Test (MPT)^27^ and the Beat Alignment Test (BAT)^28^ in the sample of 86 listeners who took part in the present study.

We first tested for covariance between the two measures of musical aptitude, beat perception as reflected in the Beat Alignment Test (BAT) index^28^ and melody perception as reflected in an index derived from the Mistuning Perception Test (MPT)^27^. The test revealed a weak positive correlation (R^2^ = 0.26, *p* = 0.016), indicating that listeners with lower beat perception ability also had lower melody perception ability (and vice versa). The correlation plot is shown in Fig. 1. Given the relatively minor degree of covariance of the two musical aptitude traits among the participants of the present study (i.e., R < 0.5), no collinearity issues were expected to apply to the statistical modelling of these data^29^.

### Song-likeness of the stimuli before and after repetition

**Fig. 2:**
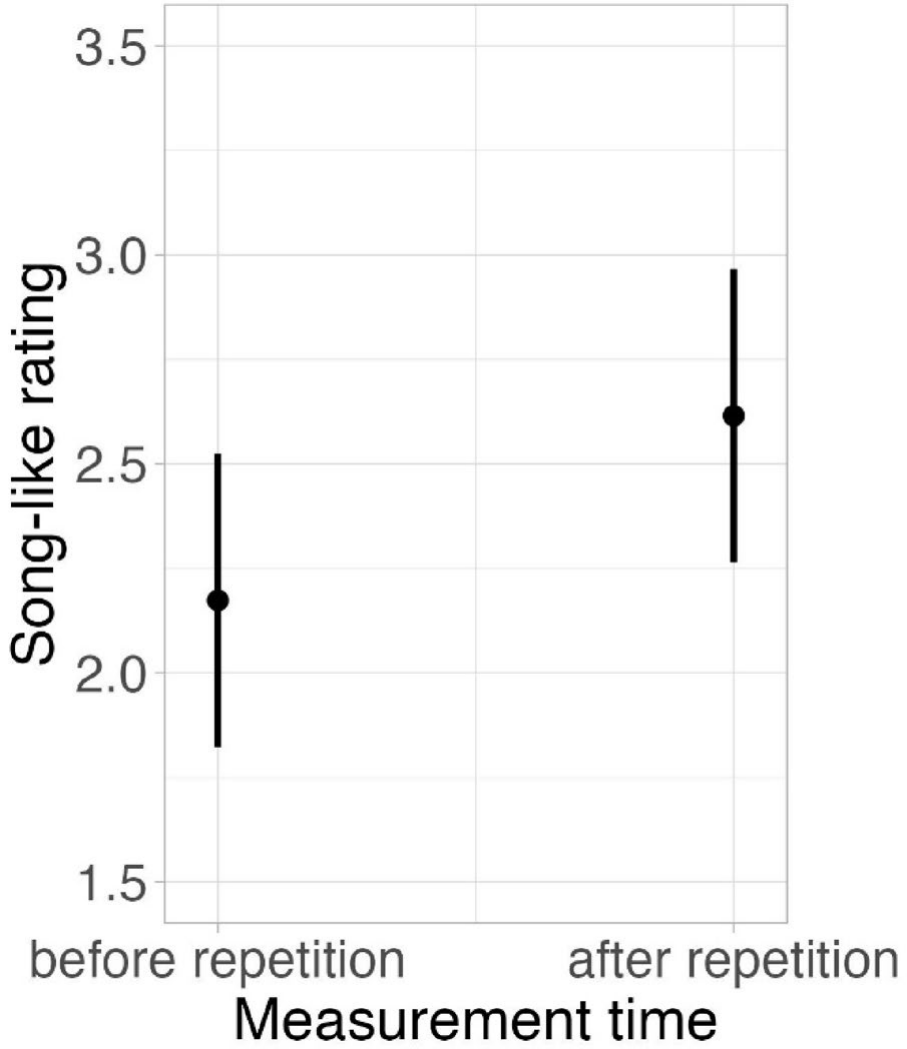
Perceptual ratings of test sentences on the song-likeness scale (1 = *clearly speech*, 8 = *clearly song*) after a single exposure (before repetition) versus after repeated exposure (after eight repetitions).

We then examined if repetitions of the stimuli created for the present study led to the experience of STS among the listeners recruited for the study. For this, we compared the song-like ratings of the stimuli collected at the beginning of the experiment (i.e., before exposure to repetitions) and at the end of the experiment (i.e., after exposure to repetitions), and identified a significant effect of repetition on increasing the song-likeness of a stimulus (χ^2^ = 37.51, df = 1, *p* < 0.001, see Fig. 2), suggestive of a successful perceptual transformation into song upon repetition^10^.

### Likelihood of the illusion

**Fig. 3:**
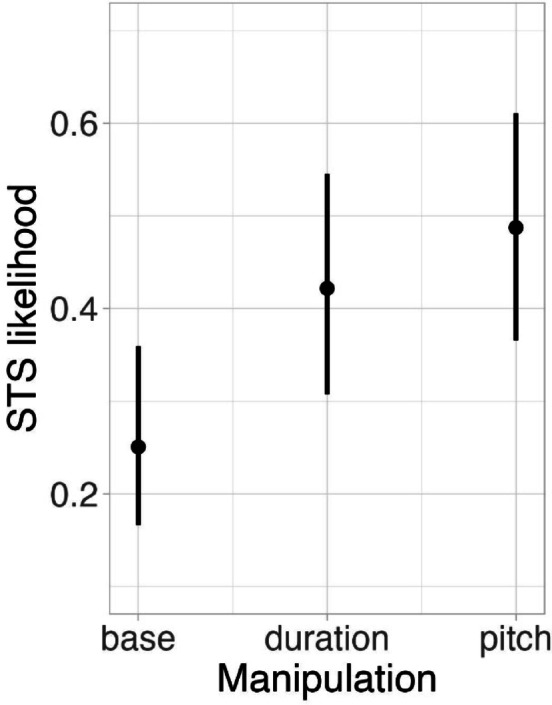
Model estimate plot obtained from the best-fit model of the likelihood of the speech-to-song illusion (STS), displaying the main effect of manipulation (comparing stylised baseline to duration- and pitch-manipulated stimuli).

We fitted a logistic mixed-effects model to test how well experimental factors of the present study could predict both the likelihood of listeners experiencing the illusion and that of stimuli eliciting it. The best-fit model found a significant effect of the manipulation (χ^2^ = 9.29, df = 2, *p* < 0.01, see Fig. 3) which indicated that both duration (β = 0.78, SE = 0.32, z = 2.46, *p* = 0.037) and pitch (β = 1.04, SE = 0.32, z = 3.28, *p* = 0.003) manipulations increased the likelihood of a stimulus to transform into song compared to baseline (i.e., a stylised but otherwise not manipulated stimulus). Stimuli of both manipulations were equally likely to transform around 50% of the time (β = 0.27, SE = 0.31, z = 0.86, *p* = 0.668). Musical aptitude of listeners was not significant either as a main effect (MPT: χ^2^ = 0.02, df = 1, *p* = 0.881; BAT: χ^2^ = 0.97, df = 1, *p* = 0.325) or in interaction with manipulation (MPT: χ^2^ = 4.50, df = 2, *p* = 0.105; BAT: χ^2^ = 4.97, df = 2, *p* = 0.083).

### Speed of the illusion

**Fig. 4:**
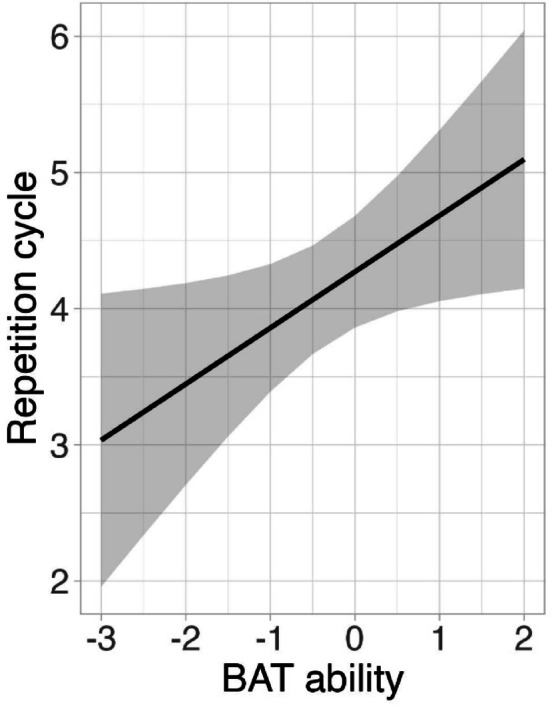
Model estimate plot obtained from the best-fit model of the speed of the speech-to-song illusion, displaying the main effect of individual beat perception ability as measured by the Beat Alignment Test (BAT)^28^. The speed of the illusion is plotted along the y-axis (the total number of repetition cycles was 8).

We fitted an ordinal mixed-effects model to examine which experimental factors would predict the occurrence of the illusion during the eight repetitions of each spoken phrase. The best-fit model retained a significant effect of beat perception ability (χ^2^ = 6.17, df = 1, *p* = 0.013, see Fig. 4), indicating a negative relationship between beat perception and the speed of the illusion (β = 0.61, SE = 0.24, z = 2.52, *p* = 0.012). That is, listeners with lower beat perception ability reported the transformation occurring, on average, 1–3 repetition cycles earlier than listeners with higher beat perception ability. Predicted interactions of individual musical aptitude traits with manipulation were not significant (MPT: χ^2^ = 0.78, df = 2, *p* = 0.678; BAT: χ^2^ = 0.54, df = 2, *p* = 0.764). There was also no main effect of either melody perception ability (χ^2^ = 0.01, df = 1, *p* = 0.930) or manipulation (χ^2^ = 1.94, df = 2, *p* = 0.379) on the speed of the illusion.

### Strength of the illusion

**Fig. 5:**
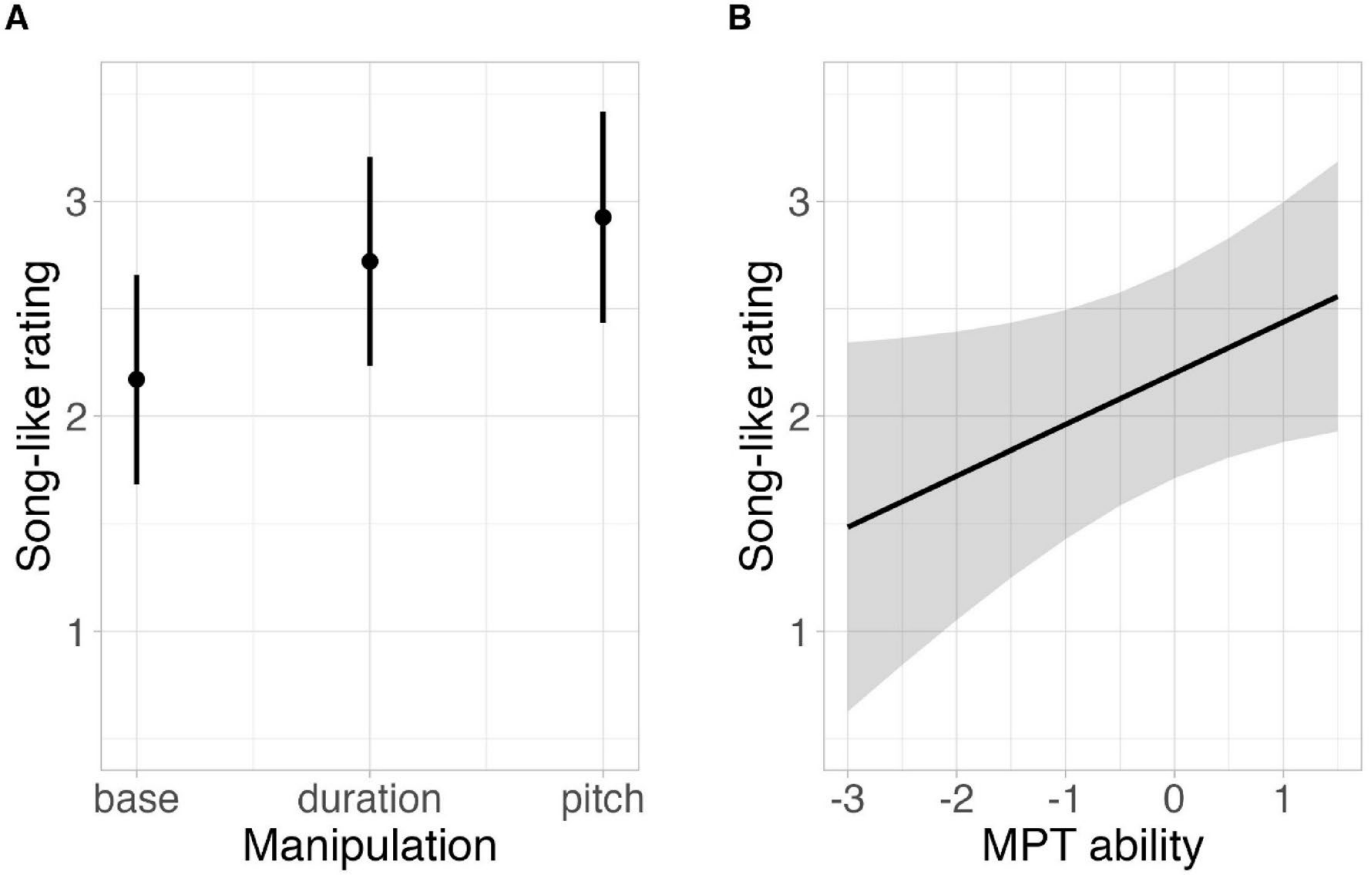
Model estimate plot obtained from the best-fit model of the strength of the speech-to-song illusion, displaying the main effects of (**A**) stimulus manipulation and (**B**) individual melody perception ability as measured by the Mistuning Perception Test (MPT)^27^. Song-like ratings (on an 8-point scale from 1 = *clearly speech* to 8 = *clearly song*) were collected after exposure to stimulus repetitions.

We fitted an ordinal mixed-effects model to test for the effect of any experimental factors on the strength of the illusion. The best-fit model retained two main effects, manipulation (χ^2^ = 7.18, df = 2, *p* = 0.028, see Fig. 5A) and melody perception ability (χ^2^ = 4.39, df = 1, *p* = 0.036, see Fig. 5B). The main effect of manipulation indicated that pitch-manipulated stimuli sounded more song-like after repetition than stylised baseline stimuli (β = 1.20, SE = 0.44, z = 2.75, *p* = 0.017). Neither baseline versus duration-manipulated stimuli (β = 0.91, SE = 0.43, z = 2.10, *p* = 0.089) nor the two manipulated versions of the stimuli (β = 0.29, SE = 0.43, z = 0.68, *p* = 0.776) differed significantly in the illusion strength. There was a positive relationship between the reported strength of the illusion and individual melody perception ability, indicating that listeners with higher melody perception ability experienced the stimuli more song-like after repetitions than listeners with lower melody perception ability (β = 0.44, SE = 0.21, z = 2.11, *p* = 0.035). Predicted interactions of musical aptitude traits with manipulation were not significant (MPT: χ^2^ = 5.16, df = 2, *p* = 0.076; BAT: χ^2^ = 0.544, df = 2, *p* = 0.762). There was also no effect of beat perception ability (χ^2^ = 0.129, df = 1, *p* = 0.720) on the strength of the illusion.

## Discussion

The present study was conducted to test the interplay between acoustic (i.e., stimulus-related) and perceptual (i.e., listener-related) underpinnings of the speech-to-song (STS) illusion^1,2^, with the goal of advancing current understanding of how and why the illusion arises in a listener’s mind. Previous work suggests that individual perceptual sensitivity to musical characteristics and the latent presence of such characteristics in spoken phrases work synergistically during the transformation from speech to song^23^. Following on from this proposal, the present study manipulated pitch and duration of otherwise low-transforming spoken phrases^19^ and expected to find an interaction of stimulus manipulation and listeners’ musical aptitude traits during repetitions, hypothesising that listeners with high melody perception ability would experience STS more frequently, faster, and more vividly in pitch-manipulated phrases while listeners with high beat perception ability would experience STS more frequently, faster, and more vividly in duration-manipulated stimuli. The results provide only partial support to these hypotheses as no hypothesised interaction was found significant in the data collected from 84 participants who took the study online, and the finding for beat perception ability was in the direction opposite to the predicted effect^23^.

Turning first to the manipulations of pitch and duration in the stimuli, which followed the rationale of our past studies^7,19^, we found that both manipulations successfully contributed to an increased transformability of otherwise low-transforming^19^ spoken phrases. Stimuli with either type of manipulation showed significantly higher rates of transformation into song compared to stylised but otherwise unmanipulated baseline stimuli. Stable pitch trajectories and long periods of high sonority are known to be the defining features of song acoustics^3,4,30^, and may therefore serve as reliable acoustic cues to setting a strong perceptual bias toward singing during repetitions^13^. The likelihood of experiencing the illusion was not moderated by either melody or beat perception ability, suggesting that in simple short phrases like the ones used in the present study, the presence of a strong acoustic cue is generally sufficient to induce the illusion upon repetitions, even in casual music listeners without distinguished musical aptitude^17^. As previously suggested, knowledge of acoustic differences between speech and song can be acquired implicitly, through everyday music exposure and without directed instruction or training^18^. Such implicit knowledge has been hypothesised to play a crucial role in biasing perception toward song and thus facilitating the illusion^13,17^. In the absence of strong acoustic cueing, individual listener traits may, however, regain their relevance in influencing transformability of spoken phrases into songs as indicated in previous studies^13,31^.

With regard to the speed of the transformation, only individual beat perception ability showed a systematic effect on how quickly the percept of a song arose in a listener’s mind during repetitions. The effect pointed in the direction not predicted by the musical aptitude hypothesis^23^, given that listeners with lower beat perception ability reported experiencing the transformation into song earlier than listeners with higher beat perception ability. Transformation speed—indicative of the ease with which the speech percept is released and the song percept arises^13^—was not tested in previous research into the role of music aptitude in STS^23^, even though it consitutes one of the key aspects of individual experience of the illusion^13,19^. We interpret the present finding as being supportive of the idea that goes back to the discovery of STS by Diana Deutsch who originally suggested that some kind of a perceptual distortion must happen during repetitions before the mind can experience speech as song^2^. In extension—or revision—of the original idea that repetitions make the pitches become distorted such that they conform to a well-formed melody^2^, the present finding indicates that the distortion may be primarily rhythmic rather than melodic in nature. Repetition is known to foreground rhythmic elements of acoustic signals—an effect which has been widely exploited in music^10,12^. Relatively poor perceptual encoding of beat timing in listeners with low beat perception ability may lead to a fast individual experience of the loop as being more rhythmic than it actually is. In contrast, relatively good perceptual encoding of beat timing in listeners with high beat perception ability appears to preserve the veridical percept of temporal relationships in spoken phrases, delaying the transformation in the mind of those listeners. We suggest that a temporal distortion as to establish perceptual rhythmicity and a steady beat consitutes the first step in the transformation from speech to song during repetitions. This suggestion is in keeping with the finding that the illusion is experienced faster by non-native than by native listeners^19^, given that perceptual encoding of speech timing in native speech is superior to that of non-native speech^32^.

Finally, the strength of the illusion—or the vividness of a song percept in the listener’s mind—showed the predicted effect of stimulus manipulation and individual melody perception ability, though without an interaction. Our stimulus manipulation replicated a strong illusion-inducing effect of stable pitch, confirming its central role in the experience of STS^7,20^. In line with the musical aptitude account of STS^23^, participants with higher melody perception ability experienced the illusion more strongly after being exposed to repetitions of spoken phrases. This equally applied to all stimuli of the study and not just to those that were manipulated in pitch, indicating that the effect may be more general than previously suggested^23^. Experience of the illusion is strongly linked to an increased perceptual salience of pitch, marked by a heightened activation of pitch-sensitive cortical areas^9^. In contrast to previous musical aptitude accounts of the illusion, we do not think that a perceptual distortion of pitch is at play in listeners with high melody perception ability^2,23^. Rather, we suggest that listeners who are well attuned to pitch in musical melodies are also better able to access musical versions of increasingly salient pitch contours in speech^7^. These listeners may store a larger repertoire of musical tunes at their perceptual disposal, which also equips them well for high performance on the musical mistuning test used in the present study^27^. This interpretation is in line with neuroimaging evidence suggesting that differential brain responses to song versus speech cannot be attributed to low-level acoustic processing but arise more likely from a higher-level ability to process musical regularities embedded in the song stimuli^9^. We interpret the lack of an interaction with the manipulated stimulus acoustics as an indication that the individual ability to access musical pitch representations does not depend on strong acoustic cueing. However, the effects are likely additive, suggesting that the most vivid song percept occurs when stable-pitch stimuli are experienced by listeners with high melody perception ability.

Taken together, the findings of the present study resonate with the idea that auditory processing of time and pitch may be dissociable to a certain extent^33^ and that their role in the perception of speech and music differs also^34,35^. As indicated in previous research, speech perception requires high temporal resolution while music perception crucially hinges on high frequency resolution^34,35^. There is some evidence for a functional asymmetry in which left auditory cortex specialises primarily in rapid temporal processing and right auditory cortex in frequency resolution, providing a framework for hemispheric differences in speech and music processing, potentially linked to anatomical asymmetries in cortical myelination and columnar organisation^35^. This aligns well with the findings of a study into the neural circuitries involved in the perception of the illusion^9^. Using functional MRI, the study found that early auditory regions (e.g., primary auditory cortex) did not show differential activation in response to high-transforming than to low-transforming spoken phrases; the distinction emerged later in the processing stream. A specific network of eight brain regions (most notably including the anterior superior temporal gyrus and the right mid-posterior superior temporal gyrus) exhibited significantly stronger activation when speech transformed into song in contrast to when it was perceived as regular speech, and there was stronger right- than left-hemisphere activation in response to the highly transforming spoken phrases^9^. These findings indicate that perceiving speech as song places higher demands on both pitch processing and auditory-motor integration, primarily in the right hemisphere. They do, however, not demonstrate how increased activation of neural circuitries underpinning temporal processing (e.g., in the basal ganglia and the cerebellum, which support higher temporal resolution necessary for speech perception)^36,37^ may prevail in those individuals who do not experience speech as song under the STS paradigm. Clarifying the neural bases and perceptual consequences of individual variability in different aspects of STS perception (i.e., likelihood, speed, and strength of the illusion) remains an important direction for future research.

In summary, the results of the present study provide new evidence that short spoken phrases can generally induce the speech-to-song illusion, when repeated^10^. The transformation frequency is especially high for stimuli with stable pitch and long periods of high sonority^7,19,20^ and is not dependent upon musical aptitude of listeners^17^. However, other aspects of the illusory experience are moderated by invidivual musical aptitude traits. The transformation from speech to song occurs faster in listeners with lower beat perception ability, indicating involvement of temporal distortion processes during repetitions^2^. It is experienced more vividly by listeners with high melody perception ability^23^, indicating activation of musical pitch representations upon repetition^9,23^. Overall, the present study documents that repetitions tend to evoke musical percepts in otherwise non-musical sounds^10^, providing new evidence for the discussion of the origins of this striking perceptual effect spanning two cognitive systems of the human mind—language and music.

## Methods

### Participants

One hundred and seven native speakers of English volunteered to take part in the experiment, though only 94 (mean age = 41.32, range = 20–71, 37 female) completed all tasks. The participants were recruited and remunerated via the online platform Prolific Academic (www.prolific.co). They reported having received between 0 and 17 years of musical training at the time of testing, but there were no professional musicians among the volunteers. All participants confirmed that they have never received an official diagnosis of speech or language impairments and/or amusia, which were considered exclusion criteria. Upon completion of quality checks (see below), only data from a total of 86 participants were included in the analyses.

### Materials

For the speech-to-song illusion task, eleven English sentences were chosen from a database recorded for a previous study^19^. Nine of them had low sonority (i.e., they contained a high number of voiceless stops and fricatives that interrupt the transmission of pitch information)^38^ and were used as test sentences. Two of them had high sonority and were chosen as distractors. The pitch and duration manipulations were created for the nine test sentences only. The manipulations were conducted using Praat^39^. First, a stylised-pitch version of each sentence was prepared without any manipulations. This version functioned as a low-transforming baseline^19^. From the stylized version of each test sentence, we derived a pitch-manipulated version that removed local fluctuations of f0-trajectories while keeping the pitch ratios between pitch-accented syllables constant. Using the stylised version of each test sentence again, we lengthened voiced, sonorous parts of all syllables (mostly vowels) by 20% and shortening the duration of voiceless, obstruent parts of all syllables (mostly voiceless stops and fricatives) by an equal amount to keep the overall phrase duration constant. One randomly manipulated version of each sentence was further created as a set of distractors. The procedure resulted in 27 test stimuli (nine sentences in three experimental conditions—base, pitch, duration) and 11 distractors (two high-sonority unmanipulated sentences, nine low-sonority, randomly manipulated sentences). Each participant listened to two test stimuli in each condition and four distractors, i.e. to ten stimuli in total. Latin-square design was used to allocate test sentences to individual participants while distractors were chosen in a pseudo-random fashion, with each participant hearing at least one high-sonority distractor. The stimuli were looped with eight repetitions, each separated by a 400 ms pause^7,13,19^. As indicated in previous research, close proximily of repetitions following each other in time tends to increase the likelihood of the illusion^7^.

### Tasks and procedure

The experiment consisted of five tasks and was running on the online platform Gorilla (www.gorilla.sc) ^40,41^. At the beginning of the experiment, an information sheet and a consent form were presented. Once participants consented to taking part in the study, they were instructed to use a laptop computer and to play the sounds of the experiment through headphones. An audio test at the beginning of the experiment ensured that this technical requirement was met by all participants.

After the set-up check, participants had to fill in a questionnaire that inquired about their musical background, current and past involvement in a music and/or dance practice. The questionnaire also screened for the native language requirement (English), potential speech and/or language impairments, and amusia. After the questionnaire had been completed, auditory tasks began. Participants were first asked to listen to individual test sentences, played back only once, and to rate them on a scale from 1 (*clearly speech*) to 8 (*clearly song*). These ratings served as the baseline of perceived song-likeness of each sentence prior to repeated exposure^7,10,11^.

Subsequently, participants’ beat perception ability (here, defined as individual sensitivity to temporal misalignments of the beat) was tested. For the purpose of the present study, we used the Computerised Adaptive Beat Alignment Test (CA-BAT)^28^ which asked participants to identify temporal discrepancies between a metronome beat and a musical excerpt. The test adjusts the difficulty dynamically, initially presenting an easily detectable misalignment and then adapting the difficulty level based on participants’ performance. The individual BAT index obtained from this test is z-score normalised with reference to the sample of the original norming study that tested 197 participants aged between 18 and 75 years, with a mean age of 26 years^28^. A score around 0 indicates an average beat perception ability, scores above 0 suggest an above-average ability, and scores below 0 indicate a below-average ability. Previous work indicates that beat perception (here, BAT) ability as measured by CA-BAT can predict veridical perception of speech rhythm in materials of varying length and linguistic complexity^42^, in both native and non-native languages of a listener^43^.

The second musical aptitude task tested participants’ melody perception ability (here, defined as individual sensitivity to vocal mistuning, i.e., deviations from the melodic structure of a song). We used the Mistuning Perception Test (MPT)^27^ to assess this ability. The test measures sensitivity to pitch accuracy in vocal musical performance using realistic stimuli from modern pop music performances^27^. Vocal tracks of original song exerpts were pitch-shifted in 5-cent increments, comprising minimally 10- and maximally 100-cent shifts, and implementing both sharp (i.e., upwards) and flat (i.e., downwards) mistunings. Similar to CA-BAT, MPT is also an adaptive psychometric test that adjusts in difficulty depending on participants’ performance and defines the individual ability on the same metric, a z-score (typically ranging from − 4 to 4). Similar to CA-BAT, the score of 0 is derived with reference to the population mean which in case of MPT was obtained from a sample of 333 participants aged between 18 and 70 years, with a mean age of 25 years. Positive or negative values describe how many standard deviations above or below the mean an individual listener performed on the perception of mistuning. The melody perception ability as measured by MPT has been found to correlate with the ability to perceive various pitch differences in speech materials and was therefore considered a well-suited test for the goals of the present study^44^.

As the last test of the experiment, participants had to listen to the looped test sentences and were required to indicate when they heard a transformation from speech to song by pressing a button as soon as they heard it. They were clearly instructed not to press any buttons if they did not experience a change in the percept. At the end of each trial, they evaluated the song-likeness of the sentence on the same scale as in the baseline test, i.e., from 1 (*clearly speech*) to 8 (*clearly song*). From this procedure, we obtained data on the likelihood, speed, and strength of the illusion^7,13,19,31^.

The study was approved by the ethics committee of the University of Konstanz (IRB statement 05/2021, dated 04/02/2021). The experiment was conducted in accordance with relevant guidelines and regulations. All participants provided informed consent to take part in this research and received a small payment for their time (approximately 25–30 min).

### Statistical analyses

Since there were no catch trials to check if participants had paid attention to the tasks, we removed the data of those participants who performed worse than 3 standard deviations below the mean on mistuning and/or beat alignment tests. This applied to the data of eight participants in total (8.5%). Overall, participants were quite motivated in taking part. They were free to leave any comments after completing the experiment, with following statements recorded, among others—P01: *“As a side note I would just like to say that I found your study very enjoyable.”*; P02: *“It is the best study I have undertaken.”*; P03: *“Oh wow! How the hell did that spoken phrase turn into a song? That is amazing. I had never come across that phenomenon before.”*; P04: *“I can’t get it out of my head…I’ll be whistling it for the rest of the day!”;* P05: *“What is interesting is during the study I heard a spoken phrase and i thought, aye, certainly a spoken word. Score it 1…but as the study progressed so did my judgement on the continuum as I started to hear melody!”.*

The data were preprocessed and analysed in Rstudio (running R-version 4.3.1)^45^. Apart from the basic packages, statistical analyses used *ordinal*^46^*, lme4*^47^*,* and *lmerTest*^48^ libraries. We first examined if the effect of repetition was observed in the stimuli of the present study by comparing song-like ratings given to the stimuli before and after repetion in an ordinal mixed-effects model. We then tested the effects of the predictor variables—specitically, the interactions of manipulation with individual melody perception (here, MPT) ability and manipulation with individual beat perception (here, BAT) ability—on the three dependent variables of interest: (1) the likelihood of STS (i.e., whether or not participants reported having perceived the transformation, coded as 1 or 0 and modelled as a binomial mixed-effects model); (2) the speed of STS (i.e., during which of the eight repetition cycles participants reported having perceived the transformation, modelled as an ordinal mixed-effects model); and (3) the strength of STS (i.e., how song-like on a scale from 1 to 8 the stimuli sounded to participants after repetitions, modelled as an ordinal mixed-effects model). Logistic regressions were estimated using maximal likelihood and the BOBYQA optimiser^47^. Ordinal regressions deployed ordered probit models^46^.

All models further included two crossed random intercepts, listener and stimulus. We allowed for random slopes of manipulation over listener and individual musical aptitude traits over stimulus. Random slopes were only retained if the models converged. Likelihood ratio tests were used to establish the best model fit^48^. To select the final model, we implemented a stepwise backward-fitting procedure. The first model included both predicted interactions (manipulation with BAT ability and manipulation with MPT ability). We iteratively reduced the model complexity by removing those predictors that did not improve the model fit. We used *emmeans* library to conduct post-hoc factor level comparisons of the three levels of manipulation, which implements Tukey correction for multiple comparisons by default^49^.

## Data Availability

The dataset collected and analysed for the present study is available from the corresponding author on reasonable request.
